# Thermal Dependence of Optical Parameters of Thin Polythiophene Films Blended with PCBM

**DOI:** 10.3390/polym10040454

**Published:** 2018-04-19

**Authors:** Janusz Jaglarz, Anna Małek, Jerzy Sanetra

**Affiliations:** 1 Materials Engineering, Mechanical Department, Cracow University of Technology, Jana Pawła II 37 str., 31-867 Cracow, Poland; pujaglar@cyfronet.pl; 2 Department of Electronics, AGH University of Science and Technology, Mickiewicza 30 Av., 30-059 Cracow, Poland; 3 Institute of Physics, Cracow University of Technology, Warszawska 24 str., 30-841 Cracow, Poland; pusanetr@cyf-kr.edu.pl

**Keywords:** conjugated thin films, polythiophene blends, spectroscopic ellipsometry, thermo-optical investigations

## Abstract

The main purpose of this work is to show the thermal dependence of the refractive and extinction indices of conjugated polymer films used in optoelectronics devices. Herein, we present the results of optical investigations performed for the following polymers: poly(3-hexylthiophene) (P3HT), poly(3-octylthiophene) (P3OT), and their blends with [6,6]-phenyl C61 butyric acid methyl ester (PCBM). For our analysis, we chose well-known polythiophenes such P3HT and P3OT, often used in photovoltaic cells. Our addition of PCMB to the polythiophenes allows their conversion efficiency to be increased. This paper presents the results of our investigation determining the spectral dispersion of optical constants in a wavelength range of 190–1700 nm by using spectroscopic ellipsometry (SE). Furthermore, we show the temperature dependence of the refractive indices of polythiophene films for a heating and a cooling process in the temperature range 25–130 °C. Additionally, thermo-optic coefficients and an optical gap were established and are presented in the paper, followed by a discussion on the conditions of the thermal stability of polythiophene blends and reversibility issues in thermal processes. Our paper presents a new and fresh analysis of depolarization beams after their reflection from the studied films. The paper presents the results of thermo-optical studies of polymer blends which have not been included in previously published works.

## 1. Introduction

For many years, conjugated polymers have attracted the attention of researchers in the field of organic semiconductors. One of the reasons for this interest is their optoelectronic properties that make them suitable for application in organic light emitting diodes (OLEDs) [1,2,3,4] and photovoltaic (PV) cells [2,4,5,6]. The unique optoelectronic properties of π conjugated polymers are the result of π bonds in the electronic structure of the polymer [7]. Because π bonds are not strong, it is possible to excite electrons to excited states by the photons from the visible range of the electromagnetic spectrum (VIS). For comparison, in most semiconductive polymers, σ bonds are much stronger than π bonds, and they can therefore be excited only by photons from the ultraviolet range (UV). Consequently, optical properties in the VIS region determined by π electrons and σ excitations are usually not taken into consideration [7]. The other attractive properties of conjugated polymers are the following: their easy solubility, environmental stability, and solution processability [1,4].

The effectiveness of semiconductive polymers used as LED or PV devices depends on their optical parameters, especially on the refractive index (*n*), the extinction coefficient (*k*), and the optical gap (*E*_g_) [8,9,10]. The optoelectronic properties of π conjugated polymers are a function of their chemical structure, the spatial arrangement of polymeric chains, and the film morphology [1,11,12,13]. In turn, both the polymer microstructure and the film morphology are strongly dependent on the processing conditions. It has been found that, besides the specific chemical structure, optoelectronic properties vary with changing molecular weight [5,14], the deposition method used [15], solution concentration, and film thickness [15].

For our studies we chose poly(3-octylthiophene) (P3OT) and poly(3-hexylthiophene) (P3HT) [16,17] and their blends with fullerene derivatives ([6,6]-phenyl C61 butyric acid methyl ester, PCBM). P3HT:PCBM blends have high quantum efficiency and they are a very popular research subject due to their photovoltaic applications [18,19,20]. Therefore, the authors focused their attention on examining which organic devices are the best (OLED, PV cells, polymer transistors, or even organic lasers) based on the optoelectronic properties of π bonding conjugated polymers.

To design the architecture of electronic devices such as LEDs and solar cells, it is essential to know the energetic structure of the materials used. Therefore, it is important to choose the best testing methods that provide accurate information about the energy gap and the electronic structure of polymer films. So far, the value of the energy gap of tested materials has been deduced based on the dispersion of the extinction coefficient determined by spectrophotometric measurements using Tauc or Cody models [9,21].

The most sensitive optical method allowing the determination of the optical parameters of the films and their thickness is spectroscopic ellipsometry (SE). The SE allows the determination of the most important quantities of the electronic structure of polymer semiconductors, carried out in the widest possible spectral range [8,12,22]. For layers with thickness less than 200 nm, ellipsometric techniques give the most reliable results [22]. The analysis of ellipsometric data allows establishment of the dispersion of optical constants in the range of classical optics (190–2500 nm). In turn, these dependencies allow band structure parameters to be found using appropriate optical models [11,22,23].

The interactions between a polymer thin film and incident light are expressed at a macroscopic scale through dispersive relations of the optical constants of studied layers. Namely, the extinction coefficient (*k*) and the refractive index (*n*) are related with the electronic parameters of the band structure.

Obviously, macroscopic optical indices depend on the temperature. This is called a thermo-optic effect [24,25]. In some polymer layer applications (i.e., thermo-optical switches), large changes in refractive indices upon temperature changes are the basis for these devices [24,26]. Thus, the changes in optical constants with the temperature should be considered in issues related to the design of devices based on polymer conducting films.

To date, the optical qualities of polythiophenes and P3HT/PCBM as well as P3OT/PCBM structures have mostly been studied at room temperature [10,21]. The results of these works are referred to in this section of the article. In our paper, however, we present the results of thermo-optical analysis done for P3HT/PCBM and P3OT/PCBM blends in the temperature range of 25–130 °C. Such results and their analysis have yet to be presented in the literature. The key parameter that describes the results of the study of optical qualities depending on the temperature is the thermo-optic coefficient (*TOC*)—a parameter which is not especially popular in the works of experts in the field. The authors of this article previously described the thermo-optical qualities of clean layers of P3HT and P3OT, and are refer to their results in this paper [8].

The novelty and originality of the work is related to undertaking research in the field of material optics in the field of conductive organic compounds that find application in such fields as the design and manufacture of new LED materials and photovoltaic cells. As can be seen in the literature, there are few publications available which describe changes in the optical parameters of polymeric conductive layers—in particular polythiophenes in the temperature range within which they are used. Extreme temperatures in which optoelectronic systems can work range from 20 to 100 degrees Celcius. In our work, however, as we have explained, we have expanded the measurement temperature range of blend layers to 130 °C. Such temperatures exceed the application thresholds of these materials, but allow a temperature limit to the repeatability of polymeric optoelectronic components to be found.

An innovative aspect of this work is the analysis of the thermal stability of polythiophenes and their blends based on the depolarization of reflected beams. This type of approach has not been used before and constitutes a significant added value in terms of the experiments conducted and the method used. Based on the available literature, our work is a pioneer in the study of the thermo-optical properties of polythiophenes. The temperature hysteresis of optical coefficients for conjugative polymers has not been presented before.

### Optical Modelling

The key macroscopic quantities determined in spectral measurements are the material constants ε_1_ and ε_2_, expressed as functions of the photons’ energy [27]. They can be transformed into refractive and extinction coefficients through simple transformations which are more common for presenting results in optical ranges [22,28,29].

The basic parameters of the electronic structure of the polymer layers must be determined by means of an appropriate optical model describing the band optical transitions between the excited and valence states [22,23]. For this purpose, appropriate mechanical quantum oscillators should be selected. Electronic parameters such as amplitude (*A*), broadening (*Г*), energy center (*E*_0_), and common energy gap (*E*_g_) [30] are fundamental quantities of the electronic structure of any material. They are fitted to the spectral dependencies *n*(*hν*) and *k*(*hν*), where *ν* is light frequency [23,31].

Macroscopic parameters such as ε_2_(*hν*) and *k*(*hν*) are responsible for absorption. In Expressions (1) and (2) we present the spectral dependence of the imaginary part of dielectric function ε_2_(*hν*) that occurs in the original works.

There are many dispersion relationships that combine macroscopic material parameters with their electronic structure [23]. They are based on the band theory which describes absorption in various materials.

In the case of materials that can be presented as a set of isolated, non-interacting oscillators, the classical Lorentz oscillator is the most natural model that can be applied to describe their optical properties [30]. According to the Lorentz model, the imaginary part of the dielectric function may be expressed as:

(1) $$\varepsilon_{2L}\left(h\nu \right)=\frac{A\cdot E_{0}\cdot \Gamma \cdot h\nu }{{\left(E_{0}^{2}-{\left(h\nu \right)}^{2}\right)}^{2}+\Gamma^{2}{\left(h\nu \right)}^{2}}.$$

However, in building polymers there are many resonant states resulting from their chain structure, situated in small spectral distances from each other [32]. This is a characteristic feature of disordered materials. The convolution of these oscillators causes inhomogeneous broadening of the spectral line. This phenomenon is described by the Gaussian oscillator model (GO). Conjugated polymer films can be quoted here as an example of the problem [22]. The imaginary part of the dielectric function for GO is given in the following equation:

(2) $$\varepsilon_{2G}\left(h\nu \right)=A_{n}\left(exp\left[-{\left(\frac{\left(h\nu -E_{n}\right)}{\sigma_{n}}\right)}^{2}\right]+exp\left[-{\left(\frac{\left(h\nu +E_{n}\right)}{\sigma_{n}}\right)}^{2}\right]\right),$$

where:

(3) $$\sigma_{n}=\frac{\Gamma_{n}}{2\sqrt{\ln2}}$$

$A_{n}-\;$n-th amplitude,

$E_{n}-\;$n-th center energy,

$\Gamma_{n}-\;$n-th broadening parameter.

Organic layers are modelled using Gaussian oscillators. For narrower spectral ranges, the imaginary part of the dielectric function ε_2G_ can be described by the first exponent appearing in Equation (2).

Gaussian oscillators adequately describe the optical properties of conjugated polymers in a wider spectral range (for photon energy from 0.7 to 7 eV). However, it should be underlined that both Lorentzian and Gaussian theorems are not able to determine the common optical gap for polymer films [31].

If the crystallinity of polymer films is low, then the Tauc-Lorentz model (TL) can be used in the first approximation [33,34]. The TL oscillator is used for modelling amorphous and amorphous-like thin films. Moreover, the TL model describes absorption phenomena that occur in amorphous films near the absorption edge. In the Tauc-Lorentz model, the ε_2_ introduced by Jellison and Modine [33] is given as the product of the Tauc function ε_2TL_ and the Lorentz oscillator function *L*(*E*):

(4) $$\varepsilon_{2\mathrm{TL}}\left(h\nu \right)=G{\left[\frac{h\nu -E_{g}}{h\nu }\right]}^{2},$$

where: hν is the photon energy, *E*_g_ is the optical gap, and *G* stands for constant parameters. 

## 2. Materials and Methods

In the present work, P3HT, P3OT, and PCMB solutions purchased from Sigma Aldrich (St. Louis, MO, USA) [35] were used for the preparation of polymer and polymer blend layers. The blends of P3HT:PCBM, P3OT:PCBM, and P3HT:P3OT:PCBM (1:1 *w*/*w*, 10 mg/mL for polymer) were dissolved in chloroform. The polymer solid layers were deposited by a spin coating technique onto crystalline silicon substrates using spin coater model SCV-15 (LOT-Oriel GmbH, Darmstadt, Germany) with aligned rotational speed 1500 rotations per minute. Then, the layers were annealed in a vacuum for 30 min at the temperature of 60 °C. The crystalline silicon was applied as a substrate due to its well-defined thermo-optical properties in a wide temperature range [36]. Moreover, a higher refractive indices difference (Δ*n*) on the layer-substrate interface enhances the signal-to-noise ratio of the experimental data and thereby increases the sensitivity of the ellipsometric measurement [8,37]. For a polythiophene-Si interface, Δ*n* is about 1.8 for a wavelength of 633 nm, while for a polythiophene-glass interface it is about 0.5.

The optical and electronic properties of conjugated polymer thin films were investigated using ellipsometric spectroscopy. In this technique, changes of the light polarization due to its reflection at a surface were measured. The experimentally recorded data were ψ and Δ (Psi and Delta), which are angles defining the ratio of the amplitude of Fresnel reflection coefficients *r*_p_ and *r*_s_ components, parallel and perpendicular to incidence plane of the light, respectively.

(5) $$\frac{r_{p}}{r_{s}}=exp\left(i\Delta \right)\cdot \tan\Psi$$

The angle Δ is a phase shift between both waves.

Ellipsometric parameters Ψ and Δ are complex functions of the optical parameters of the layers (*n*, *k*), thickness (*d*), the angle of incidence (θ_i_), and the wavelength (λ) of the light:

(6) $$\{\begin{array}{c} \Delta =\Delta \left(n,k,\lambda ,d,\theta_{i}\right)\\ \Psi =\Psi \left(n,k,\lambda ,d,\theta_{i}\right) \end{array}$$

Based on a single ellipsometric measurement performed for selected angles of incidence, one system of equations is obtained. Solving the above equation, two unknown parameters can be found, (i.e., *n* and *k*). Performing measurements for *m* angles of incidence, it is theoretically possible to determine 2*m* unknown parameters. In reality, the number of determined parameters is lower, but the accuracy and reliability of simultaneous fittings of an optical model to experimental data performed for many incidence angles are much higher. Spectral dependences of ellipsometric angles (i.e., ψ(λ) and Δ(λ)) were measured in the spectral range 300–1700 nm by using a variable-angle spectroscopic ellipsometer M-2000 manufactured by J.A.Woollam Co. Inc. (Lincoln, NE, USA). The measurements were performed in air for incident angles 60°, 65°, and 70° at room temperature. The selected range of incident angles was near the effective Brewster angle of thin polymer films. This is important because the largest changes in polarization occur when the incident angle is close to the Brewster angle, where the ellipsometric measurements are most sensitive [38].

To analyse the data, we combined all angular spectra and fitted all data simultaneously. The data were analysed using CompleteEASE 5.2 software.

By means of the in situ ellipsometric (ISE) technique, temperature dependences of Psi and Delta angles were measured at the incidence angle of 70°. Temperature was changed during the heating process from 25 to 130 °C and then cooled down to the starting level. The temperature step was 10 °C, and after heating the temperature was stabilized for 5 min. During the heating and cooling process, the ellipsometric angles were measured every 30 s. This procedure allowed us to determine a temperature hysteresis loop. During all experiments, the reflected light intensity and depolarization coefficients (*D*) were measured simultaneously [14].

*D* is a ratio of the incoherent component of reflected light (*R*_Inc_) to the total light reflected from the sample ($R_{\mathrm{Tot}}$):

(7) $$D=\frac{R_{\mathrm{Inc}}}{R_{\mathrm{Tot}}}=\frac{R_{\mathrm{Dep}}}{R_{\mathrm{Tot}}}.$$

Basing on depolarization measurements, a quality assessment of the studied samples is possible.

The temperature dependence of refractive index is described using the thermo-optic coefficient (*TOC*), which is the derivative of the refractive index upon temperature *dn*/*dT* [8,24,25,39]. According to Prod’homme’s theory [40], the temperature variation of the refractive indices *n* results from the change in polarizability of the electron cloud and the density of the material changing with the temperature [41].

(8) $$\frac{dn}{dT}=f\left(n\right)=\frac{(n^{2}-1)\left(n^{2}+2\right)}{6n}\left(\Phi -3\alpha \right),$$

where α is the linear thermal expansion coefficient, φ is the temperature coefficient of the electronic polarizability, defined as: Φ = *P^−^*^1^ * dP/*dT*, where *P* is mean polarizability. Equation (8) shows the refractive index increasing with temperature in a case where the electronic polarizability term dominates. In turn, *TOC* is negative when the thermal expansion term is dominant. For most polymer materials, *dn*/*dT* strongly depends on the volume thermal expansion term, and it is higher than Φ.

## 3. Results and Discussion

**Figure 1:**
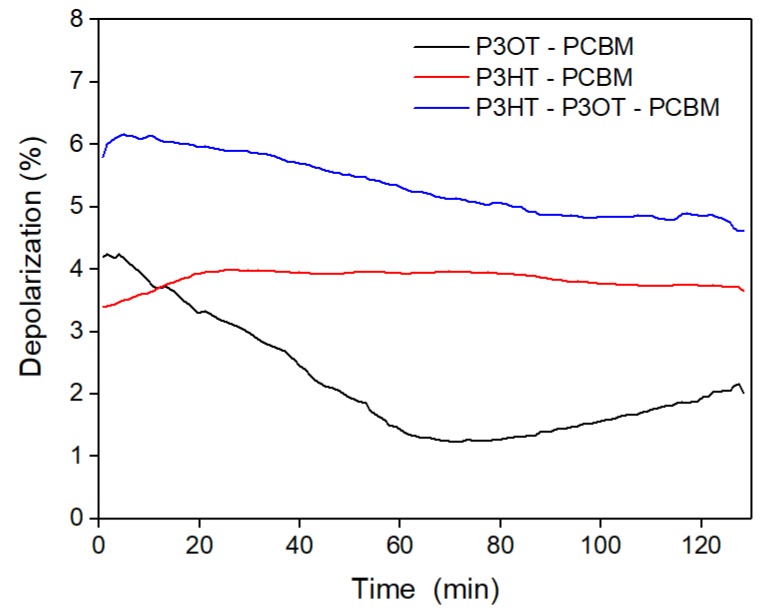
Depolarization degree of reflected light for wavelength λ = 900 nm versus annealing time. P3HT: poly(3-hexylthiophene); P3OT: poly(3-octylthiophene); PCBM: [6,6]-phenyl C61 butyric acid methyl ester.

Measurements of depolarization degree gave us insight into the homogeneity, bulk variation, and microstructure of presented films [7,15]. Moreover, we estimated the quality of polymer layers in the initial state and in heating and cooling processes. Figure 1 shows changes of depolarization coefficients determined for a light wavelength of 900 nm as a function of time of heating and cooling processes. Photons with energy corresponding to this wavelength were outside of the absorption area. The quality of the received polymer layers was assessed as fine.

Moreover, the value of the depolarization coefficient depends on the variation of the refractive thin film’s refractive index. This effect is caused by various crystalline phase fractions embedded in the amorphous medium. It is obvious to expect that a higher degree of crystallinity will give rise to a higher depolarization. In our case, the analysis of depolarization is justified because (assuming low film roughness) the higher depolarization value can partly come from crystallites randomly oriented in the film bulk and partly from the optical anisotropy of entire films. The depolarization coefficients are presented in Figure 1. The strong non-directional scattering observed in the reflection (discussed in detail here) assured us that in the main part of the layers there was an amorphous phase mixed with a smaller amount of polycrystalline phase. Thus, films can be represented by a single refractive index and an extinction coefficient. As is shown in Figure 1, the largest depolarization was exhibited by the ternary P3HT-P3OT-PCMB sample. The most thermally stable film was P3HT-PCMB, for which the depolarization coefficient was practically constant in the whole heating and cooling process.

**Figure 2:**
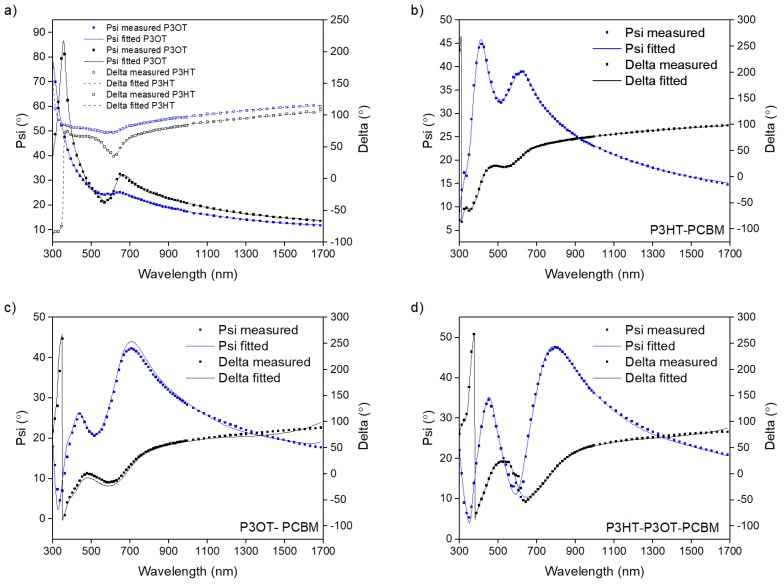
Spectral dependence of ellipsometric angles measured for (**a**) P3OT and P3HT; (**b**) P3HT/PCBM blend; (**c**) P3OT/PCBM blend; and (**d**) P3HT/P3OT/PCBM blend thin films. The results were recorded at an incidence angle of 70°.

**Figure 3:**
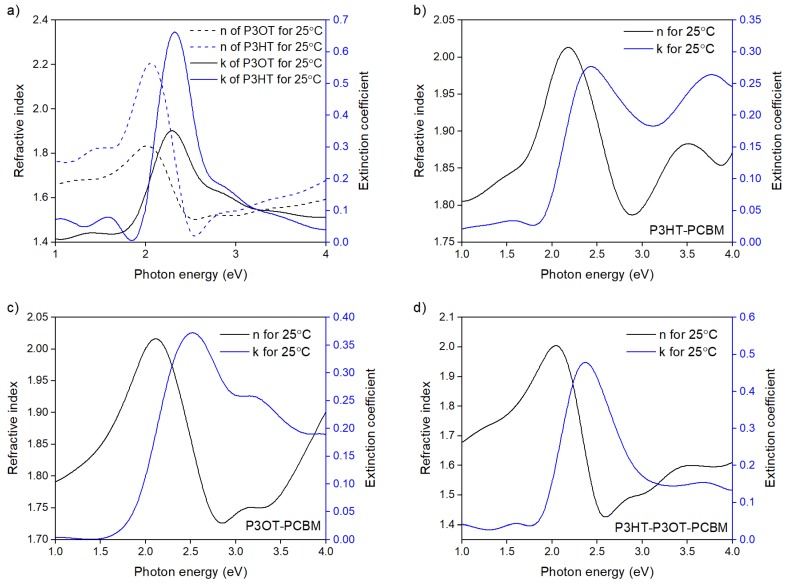
Dispersion of refractive index and extinction coefficient obtained at 25 °C for (**a**) pure P3OT and P3HT; (**b**) P3HT/PCBM blend; (**c**) P3OT/PCBM, blend and (**d**) P3HT/P3OT/PCBM blended thin films.

In Figure 2a–d we present Psi and Delta angles versus light wavelength obtained from ellipsometric study for incidence angle 70° carried out in a wide spectral light wavelength range of 190 to 1700 nm for blended polymer films. In the first fit, we chose an optical homogenous film model for the studied films. Additionally, we assumed *K*-*K* consistency in a full measured spectral range. It is shown in Figure 2 that the obtained fits were very good. However, from the practical point of view, the most important spectral range for conjugated polymer is the VIS region, which is also an area of abnormal dispersion of the presented films. We present the dispersion of optical constants *n*(*hν*) and *k*(*hν*) for polythiophene films within the photon energy range of 1 to 4 eV in Figure 3.

The light absorption occurred in range of photon energy from 1 to 3 eV. In this region, the area of the square dependence of the imaginary part of the dielectric function vs. photon energy can be found. This procedure was used to determine the optical gaps of the examined films.

All absorption maxima for polymer films were within the range of 2.5 to 2.6 eV. The broad peaks visible in Figure 3 in *k*(*hν*) spectra are associated with the heterogeneous Gaussian scattering. For polymers in the pure form (i.e., P3HT and P3OT), maximum values were lower by about 0.1 eV, while blending polymers shifted the maxima towards the larger photons’ energies. Moreover, the values of the refractive index of P3HT and P3OT layers were greater than their polymer blends.

**Figure 4:**
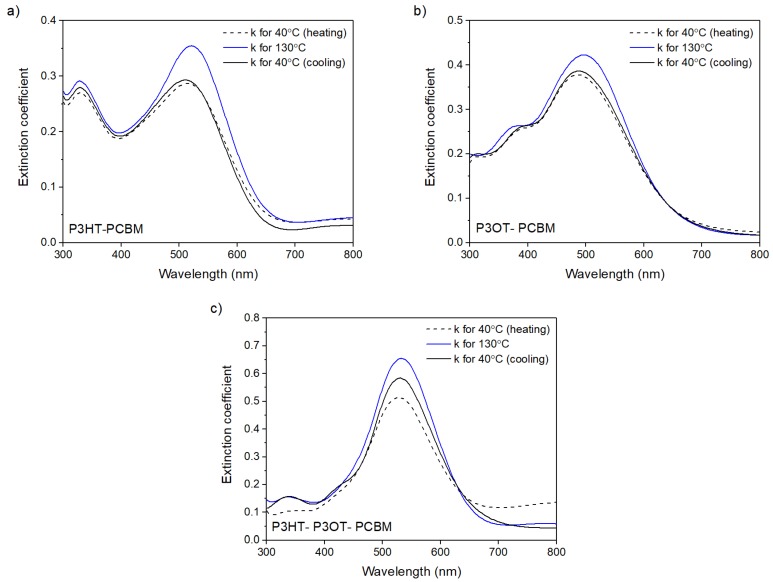
Dispersion of extinction coefficient determined for polymer blends (**a**) P3HT/PCBM; (**b**) P3OT/PCBM; and (**c**) P3HT/P3OT/PCBM at different temperatures during heating and cooling of the samples.

Figure 4 shows the spectral dependence of the extinction coefficients of polythiophene blend films vs. light wavelength for different temperatures of heating and cooling process, namely spectral dependences of *k*(λ) for initial, maximum, and final temperatures of heating and cooling processes (40, 130, and 40 °C, respectively). Values of extinction peaks in Figure 4 for all blended layers were definitely higher for the maximum temperature of the thermal process. In addition, for a higher temperature, the peaks of maximum dispersion dependence *k*(λ) moved towards shorter wavelengths. The final values of *k*(λ) were close to the initial ones for binary P3HT-PCBM and P3HT-PCBM blends. However, for a ternary P3HT-P3OT-PCBM layer, the final value of *k*(λ) was much higher than for the starting ones. Furthermore, the blended films exhibited lower refractive indices as well as lower extinction coefficients than pure P3HT and P3OT polythiophenes. The refractive and extinction coefficients for selected wavelengths and thickness of films are presented in Table 1.

Using Tauc-Lorentz and Gaussian oscillators in the fitting procedures, we determined the optical gaps, which are shown in Table 2.

The optical gap for P3HT was 2 eV, which was larger than that for P3OT (−1.8 eV). There was an energy gap of about 3.4 eV for the pure fullerene PCBM. Values of the dielectric function varied as well, but within a fairly narrow spectral region. Authors of publications in the field of conjugated polymers focus only on the absorption spectrum of polythiophenes, neglecting strong bulk and surface scattering losses [42]. This is the where our research is of great value, as we have undertaken this problem.

The key to understanding reversible processes in polymeric conductive layers is the glass transition temperature (*T*_g_). For all the studied polymers and their blends, *T*_g_ ranged from 25 to 130 °C for the bulk pure materials. We planned to carry out the thermo-optical investigation below the glass transition temperature. Therefore, to avoid a phase transition in the polymer structure, the lower range of temperature was selected for thermo-optical studies. In a reversible process, we should obtain closed thermal hysteresis for the heating and cooling of pure and mixed polythiophenes. Values of *T*_g_ for thin films could be even lower than for bulk material. Moreover, in metallic alloys, the melting temperature can be lowered by adding a new component. Consequently, the proportion of new organic components in the polymer layers decreases the glass transition temperature.

For deeper insight into the thermo-optical properties of pure and blended polythiophene layers, we analysed in situ thermal ellipsometric investigations.

**Figure 5:**
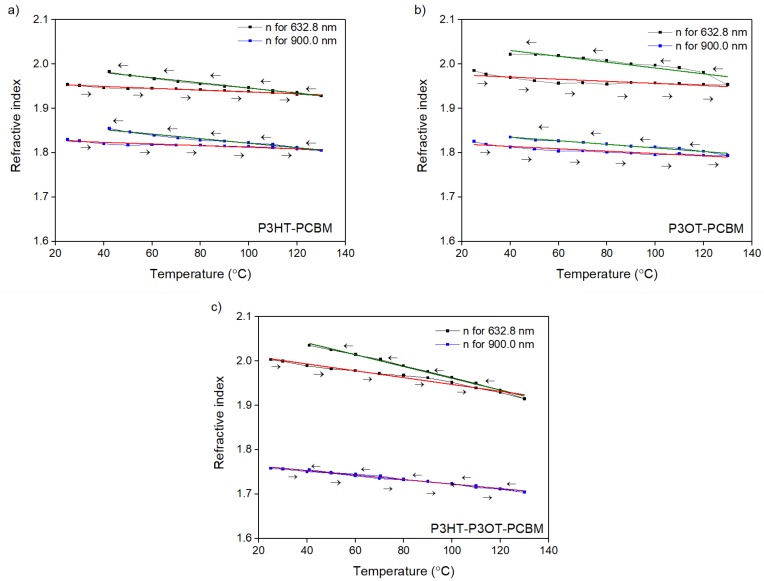
Temperature dependencies of refractive index for (**a**) P3HT/PCBM; (**b**) P3OT/PCBM; and (**c**) P3HT/P3OT/PCBM are presented for two wavelengths: 632.8 and 900 nm.

Figure 5 presents temperature dependences of polythiophene film refractive indices for 632.8 and 900 nm light wavelengths.

The linear dependencies were fitted for heating and cooling time of thermal treatments. The slopes seen in Figure 5 are the thermo-optic coefficients of refractive index (*TOC*). The values of these coefficients for selected wavelengths are shown in Table 1.

The values of *TOC* for heating and cooling processes of the polythiophene films were determined in a temperature range from 40 to 130 °C, and they are shown in columns 5 and 6 of Table 1. All *TOC*s had negative values. This behaviour is characteristic for the materials for which the temperature dependence of the optical indices is associated with thermal expansion [43].

**Figure 6:**
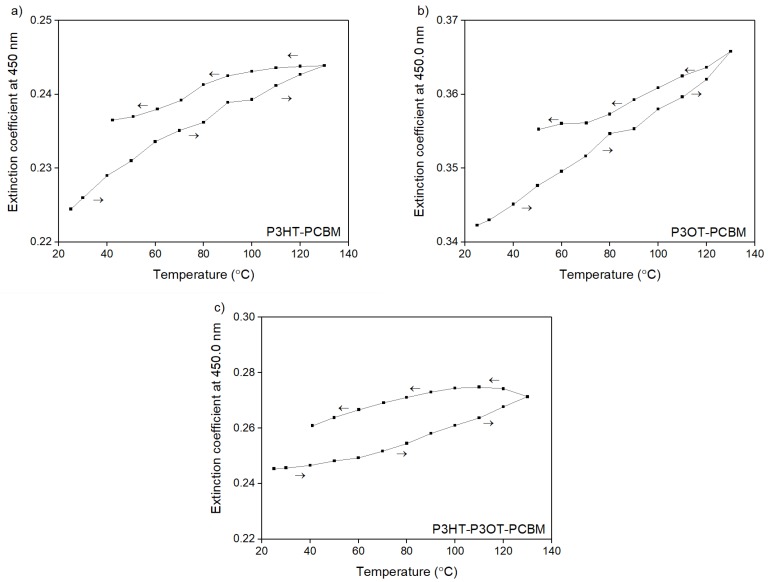
Temperature dependencies of extinction coefficient for (**a**) P3HT/PCBM; (**b**) P3OT/PCBM; and (**c**) P3HT/P3OT/PCBM blend films determined for the wavelength 450 nm.

In order to fully evaluate the repeatability of the thermo-optical process, thermal hysteresis loops for both *n* and *k* indices should be discussed. Figure 6a–c shows hysteresis loops of extinction coefficients *k* for polymers and their mixtures.

It is visible that after cooling down, optical parameters did not return to their initial values. The values of *n* and *k* indices, which form unclosed loops, are presented in Figure 5 and Figure 6. They did not return to the initial values—the hysteresis loops are open for all of them. This means that the thermal treatment in the temperature range 25 to 130 °C is definitely irreversible.

## 4. Conclusions

Our work presents a new approach to the analysis of optical results through depolarization measurements carried out in situ during the heating and cooling processes of blended polythiophene thin films. Our work proved the usefulness of this research technique. Temperature dependences of optical constants have been presented in the form of temperature hysteresis. Moreover, the analysis of the electron structure of polythiophenes films mixed with PCMB allowed us to determine optical and electronic parameters obtained from ellipsometric investigations. Thermal processes were carried out for conjugated polymer films by means of spectroscopic ellipsometry investigations.

The optical gaps for the studied films were established using Tauc-Lorentz and Gaussian oscillators in the fitting procedures.

To evaluate repeatability of the thermo-optical process, thermal hysteresis loops of optical indices were determined. Moreover, the *TOC*s for the heating and cooling processes of the polythiophene films were determined in a temperature range from 40 to 130 °C.

The study of the temperature dependence of optical constants could be used in the design of new LEDs and photovoltaic materials. In our work, we pay special attention to a qualitative and easily interpretable analysis of ellipsometric results.

A very interesting part of the work is the analysis of the depolarization of light beams reflected from layered systems containing pure or mixed polythiophenes. The conclusions that we have drawn from the analysis of the dependence of the degree of depolarization of light reflected for increasing temperatures are innovative and have not been published previously. The method of temperature analysis using the dependence of changes in the depolarization coefficient of the beam reflected from the polymer layers has not been previously described. In addition this method could be used to test new, divine materials.

## Figures and Tables

**Table 1:** Thermo-optical parameters of P3HT, P3OT, and their blends with PCBM. TOC: thermo-optic coefficient.

Sample	λ (nm)	*n*	*k*	*TOC* (10^−4^/K)		Thickness (nm)
				Heating	Cooling	
P3HT	632.8	2.15	0.061	---	---	75
P3OT	632.8	1.83	0.120	---	---	54
P3HT/PCBM	450	1.81	0.224	---	---	130
	632.8	1.95	0.056	−2.1	−5.8	
	900	1.83	0.029	−1.9	−5.1	
P3OT/PCBM	450	1.74	0.330	---	---	101
	632.8	2.02	0.090	−1.3	−7.3	
	900	1.83	3 × 10^−4^	−2.0	−4.1	
P3OT/P3HT/PCBM	450	1.44	0.261	---	---	84
	632.8	2.04	0.176	−7.7	−13.3	
	900	1.76	0.049	−5.0	−5.5	

**Table 2:** Values of energy gaps and their temperature gradients determined for studied polymer blends.

Sample	*E*_g_ (eV)	*dE*/*dT* 10^−^^3^ (eV/K)	
		Heating	Cooling
P3HT	2.02	−1.91	---
P3OT	1.79	−1.26	---
P3HT–PCBM	2.11	−1.06	0.99
P3OT–PCBM	1.92	−1.46	1.13
P3HT–P3OT–PCBM	1.99	−0.91	0.78

## References

[1] Botiz I., Stingelin N. (2014). Influence of Molecular Conformations and Microstructure on the Optoelectronic Properties of Conjugated Polymers. Materials.

[2] Hildner R., Köhler A., Müller-Buschbaum P., Panzer F., Thelakkat M. (2017). π-Conjugated Donor Polymers: Structure Formation and Morphology in Solution, Bulk and Photovoltaic Blends. Adv. Energy Mater..

[3] Tremel K., Ludwigs S. (2014). Morphology of P3HT in Thin Films in Relation to Optical and Electrical Properties. Adv. Polym. Sci..

[4] Facchetti A. (2010). π-Conjugated polymers for organic electronics and photovoltaic cell applications. Chem. Mater..

[5] Katsouras A., Gasparini N., Koulogiannis C., Spanos M., Ameri T., Brabec C.J., Chochos C.L., Avgeropoulos A. (2015). Systematic Analysis of Polymer Molecular Weight Influence on the Organic Photovoltaic Performance. Macromol. Rapid Commun..

[6] Xiao S., Zhang Q., You W. (2017). Molecular Engineering of Conjugated Polymers for Solar Cells: An Updated Report. Adv. Mater..

[7] Kobayashi T., Naito H., Singh J. (2006). Optical properties of Organic Semiconductors and Applications. Optical Properties of Condensed Matter and Application.

[8] Jaglarz J., Nosidlak N., Wolska N. (2016). Thermo-optical properties of conducted polythiophene polymer films used in electroluminescent devices. Opt. Quantum Electron..

[9] Al-Ibrahim M., Roth H.-K., Schroedner M., Konkin A., Zhokhavets U., Gobsch G., Scharff P., Sensfuss S. (2005). The influence of the optoelectronic properties of poly(3-alkylthiophenes) on the device parameters in flexible polymer solar cells. Org. Electron..

[10] Chochos C.L., Choulis S.A. (2011). How the structural deviations on the backbone of conjugated polymers influence their optoelectronic properties and photovoltaic performance. Prog. Polym. Sci..

[11] Vezie M.S., Few S., Meager I., Pieridou G., Dörling B., Ashraf R.S., Goñi A.R., Bronstein H., McCulloch I., Hayes S.C. (2016). Exploring the origin of high optical absorption in conjugated polymers. Nat. Mater..

[12] Roncali J. (2007). Molecular Engineering of the Band Gap of π-Conjugated Systems: Facing Technological Applications. Macromol. Rapid Commun..

[13] Grigorian S., Joshi S., Pietsch U. (2010). Temperature-dependent structural properties of P3HT films. IOP Conf. Ser. Mater. Sci. Eng..

[14] Koynov K., Bahtiar A., Ahn T., Cordeiro R.M., Hörhold H.-H., Bubeck C. (2006). Molecular Weight Dependence of Chain Orientation and Optical Constants of Thin Films of the Conjugated Polymer MEH-PPV. Macromolecules.

[15] Joshi S., Grigorian S., Pietsch U., Pingel P., Zen A., Neher D., Scherf U. (2008). Thickness Dependence of the Crystalline Structure and Hole Mobility in Thin Films of Low Molecular Weight Poly(3-hexylthiophene). Macromolecules.

[16] Osaka I., McCullough R.D. (2008). Advances in Molecular Design and Synthesis of Regioregular Polythiophenes. Acc. Chem. Res..

[17] Marrocchi A., Lanari D., Facchetti A., Vaccaro L. (2012). Poly(3-hexylthiophene): Synthetic methodologies and properties in bulk heterojunction solar cells. Energy Environ. Sci..

[18] Lee W.H., Chuang S.Y., Chen H.L., Su W.F., Lin C.H. (2010). Exploiting optical properties of P3HT:PCBM films for organic solar cells with semitransparent anode. Thin Solid Films.

[19] Kaur M., Gopal A., Davis R.M., Heflin J.R. (2009). Concentration gradient P3OT/PCBM photovoltaic devices fabricated by thermal interdiffusion of separately spin-cast organic layers. Sol. Energy Mater. Sol. Cells.

[20] McNeill C.R., Greenham N.C. (2009). Conjugated-Polymer Blends for Optoelectronics. Adv. Mater..

[21] Naffouti W., Mehdi A., Nasr T.-B., Kamoun-Turki N. (2013). Morphological and Optical Studies of Poly(3-hexylthiophene) Deposited by Spin Coating on Various Substrates. Energy Environ. Focus.

[22] Campoy-Quiles M., Alonso M.I., Bradley D.D.C., Ritcher L.J. (2014). Advanced Ellipsometric Characterization of Conjugated Polymer Films. Adv. Funct. Mater..

[23] Likhachev D.V., Malkova N., Poslavsky L. (2015). Modified Tauc-Lorentz dispersion model leading to a more accurate representation of absorption features below the bandgap. Thin Solid Films.

[24] Coppola G., Sirleto L., Rendina I., Iodice M. (2011). Advance in thermo-optical switches: Principles, materials, design, and device structure. Opt. Eng..

[25] Wiechmann S., Müller J. (2009). Thermo-optic properties of TiO_2_, Ta_2_O_5_ and Al_2_O_3_ thin films for integrated optics on silicon. Thin Solid Films.

[26] Han Y.-T., Shin J.-U., Park S.-H., Seo J.-K., Lee H.-J., Hwang W.-Y., Park H.-H., Baek Y. (2012). 2 × 2 Polymer Thermo-Optic Digital Optical Switch Using Total-Internal-Reflection in Bend-Free Waveguides. IEEE Photonics Technol. Lett..

[27] Guo Y., Wang Q., Kawazoe Y. (2015). A New Silicon Phase with Direct Band Gap and Novel Optoelectronic Properties. Sci. Rep..

[28] Band Y.B. (2000). Light and Matter: Electromagnetism, Optics, Spectroscopy and Lasers.

[29] Hakoe F., Tokoro H., Ohkoshi S. (2017). Dielectric and optical constants of λ-Ti_3_O_5_ film measured by spectroscopic ellipsometry. Mater. Lett..

[30] Fujiwara H. (2007). Spectroscopic Ellipsometry: Principles and Applications.

[31] Jellison G.E., Irene E.A., Thompkins H.G. (2005). Data Analysis for Spectroscopic Ellipsometry. Handbook of Ellipsometry.

[32] Barford W. (2013). Electronic and Optical Properties of Conjugated Polymers.

[33] Modine G.E., Jellison F.A. (1996). Parameterization of the optical functions of amorphous materials in the interband region. Appl. Phys. Lett..

[34] Jellison G.E., Merkulov V.I., Puretzky A.A., Geohegan D.B., Eres G., Lowndes D.H., Caughman J.B. (2000). Characterization of thin-film amorphous semiconductors using spectroscopic ellipsometry. Thin Solid Films.

[35] Sigma Aldrich Webpage. www.sigmaaldrich.com.

[36] Azzam R.M.A., Bashara N.M. (1995). Ellipsometry and Polarized Light.

[37] Campoy-Quiles M., Etchegoin P.G., Bradley D.D.C. (2005). On the optical anisotropy of conjugated polymer thin films. Phys. Rev. B.

[38] Johs B., Herzinger C.M. (2004). Precision in ellipsometrically determined sample parameters: Simulation and experiment. Thin Solid Films.

[39] Xie H., Ng F.L., Zeng X.T. (2009). Spectroscopic ellipsometry study of thin film thermo-optical properties. Thin Solid Films.

[40] Prod’homme L. (1960). A new approach to the thermal change in the refractive index of glasses. Phys. Chem. Glasses.

[41] Gulsen G., Inci M.N. (2002). Thermal optical properties of TiO_2_ films. Opt. Mater..

[42] Jaglarz J., Marszalek K., Duraj R., Noor I.M., Arof A.K., Wolska N., Sofiani Z. (2016). Elastic scattering phenomena in thin polymer layers. Mater. Today Proc..

[43] Kang E.-S., Bae J.Y., Bae B.-S. (2003). Measurement of Thermo-Optic Coefficients in Sol-Gel Hybrid Glass Films. J. Sol-Gel Sci. Technol..

